# The Effect of Goal Setting Difficulty on Serving Success in Table Tennis and the Mediating Mechanism of Self-regulation

**DOI:** 10.2478/v10078-012-0056-y

**Published:** 2012-07-04

**Authors:** Weina Liu, Chenglin Zhou, Liu Ji, Jack C Watson

**Affiliations:** 1 Tianjin University of Sport, Department of Health & Exercise Science.; 2 Shanghai University of Sport, Office of Scientific Research.; 3 East China Normal University, School of Sports & Health.; 4 West Virginia University, Department of Sport Sciences.

**Keywords:** table tennis athletes, goal setting difficulty, self-regulation, serving success

## Abstract

Goal setting difficulty has been shown to contribute to athletic performance (Burton et al., 2000). However, the potential mediating mechanism of goal difficulty on performance is unclear. Therefore, the purpose of this study was to verify the effect of goal setting difficulty on serving success in table tennis, and determine if self-regulation is the mediating variable. The current study used serving success within a one minute period as the task, and the “Athlete’s Self-regulation in Motor Learning” as the measurement tool. The experiment was designed as a 3 (serving frequency: 20/min, 23/min, and 26/min) × 2 (serving placement: left “small triangle”, and right “small triangle”) model. Participants (N = 60) in the current study were students from a physical education school. These participants were randomly assigned into the experimental and control groups. After the intervention, differences in self-regulation (p < 0.001) and serving success (p < 0.05) between the experimental and control groups were significant. For the experimental groups, there was a significant difference in self-regulation (p < 0.001) and serving success (p < 0.05) before and after the experiment. Serving frequency had a main effect on self-regulation (F (5, 24) = 12.398, p < 0.01) and serving success (F (5, 24) = 37.601, p < 0.001). Moderately difficult goal setting contributed to athletic performance. Regression analysis using bootstrapping methods revealed that self-regulation partially mediated the relationship between the two.

## Introduction

Goal setting theory was initially developed by Locke and Latham (1994) in organizational psychology, and was used to describe achievement behaviors in industry. Goal setting is one of the most effective psychological strategies for improving performance and motivation in organizational settings (Bueno et al., 2008). Although, initial research assessing goal setting effectiveness in sport was not as consistent as in work sites, Locke and Latham (1985) indicated that the application of goal setting in sport could be better than in work settings, because different types of goals can be set in sports (Kingston and Wilson, 2009), and performance can be assessed more easily. Partially due to better methodology, goal setting research in the sport and exercise realm has become more consistent (Bueno et al., 2008).

Based on their degree of difficulty, goals can be divided into hard goals, moderately difficult goals, and easy goals. Hard goals can be classified by a need to overcome difficulty, experiencing certain frustration, and spending a lot of energy and effort. Hard goals possess an extremely high level of challenge and uncertainty, making them difficult if not impossible to achieve, even with great effort. On the contrary, easy goals can be achieved easily, without much difficulty and effort. Moderately difficult goals have some difficulty, but can often be achieved through extreme effort. Moderately difficult goals are challenging, but achievable (Jia and Dong, 2006). Locke (1991) indicated that only hard goals could lead to high performance, and established the criteria for hard goals as being able to be achieved by less than 10% of those individuals who attempt them. Locke (1991) put forward a linear relationship between goal difficulty and performance, and this view was supported by some researchers working in industry (Cox, 1994) and sport psychology (Gould, 1993). However, in their meta-analysis, Kyllo and Landers (1995) found that moderately difficult goals led to the best performances. The same conclusions have been drawn from other studies (Hall and Weinberg, 1987; Ji et al., 1998; Weinberg et al., 1985). Atkinson and Reitman (1956) found an inverse curvilinear relationship between task difficulty and performance: moderately difficult goals were associated with the most effort, while hard and easy goals resulted in less effort. Burton et al. (2000) found a preference among student athletes and Olympic athletes towards moderately difficult goals. All of these findings coincide with goal setting theory, which suggests that specific and moderately difficult goals could contribute to performance improvement. Meta-analysis has shown that the effect size of goal difficulty on performance is between 0.52 and 0.82 (Locke and Latham, 1990).

Besides the topic of goal difficulty, goal setting research has addressed the question of which individual differences (e.g., holding an entity theory versus an incremental theory of human capabilities, Dweck, 1999) or contextual factors (e.g., team structure) facilitate the development of different goals. Due to the complexity and variability of the sport context, additional investigation into individual differences may be valuable. According to the entity theory, personal attributes such as intelligence are innate, trait-like qualities that are fixed in nature and are difficult to change or develop. In contrast, individuals endorsing an incremental theory perceive that personal attributes such as intelligence are not innate, but dynamic and malleable in nature and can be developed through sustained effort and experience. As Sockett (1988) noted, there are several personal qualities individuals can develop (i.e., self-regulation, carefulness, onscientiousness, and endurance) to make their goal pursuits easier. Among the personal qualities, self-regulation has had the most attention paid to it. Latham and Locke (1991) described the self-regulatory effects of goal setting, and suggested that goal setting could be used as a self-regulatory technique. Based upon this suggestion, goal-setting has been used as a kind of self-regulatory intervention technique in some research (Fiske and Taylor, 1991; Gainforth et al., 2011; Kolovelonis et al., 2011; Miller et al., 1993). More importantly, VandeWalle et al. (1999) found that the relationship between goal orientation and sales performance was fully mediated by self-regulation tactics. Therefore, it is important to understand that goal setting can be interpreted from a self-regulatory perspective (Oettingen et al., 2000).

A theory on fantasy realization (Oettingen, 1999) has also been used to analyze goal setting by proposing different avenues of goal formation based upon different forms of self-regulatory thought (i.e., expectations versus free fantasies, solely fantasizing about a positive future, and merely reflecting on the negative reality). Experimental studies by Oettingen et al. (2001) have highlighted the value of self-regulatory strategies and suggest effective strategies for setting and implementing goals (i.e., contrasting fantasies about a desired future with present reality and forming implementation intentions, respectively). According to the self-regulatory model (Bandura and Wood, 1989), people can form self-efficacy judgments and set goals for their performance in a specific task. Bueno et al. (2008) also argued that “The effect of self-efficacy on subsequent performance is both direct and mediated by personal goals, and personal goals directly influence subsequent performance. From this perspective, it is also believed that present performance will influence subsequent self-efficacy and goal setting. Thus, the above pattern is a cyclical and dynamic process”. In the sport domain, the validity of the self-regulatory model has been confirmed (Kane et al., 1996; Theodorakis, 1995; 1996).

Based upon the aforementioned theories, in conjunction with past findings, the present investigation was designed to verify the effect of goal setting difficulty on serving success in table tennis, and determine if self-regulation serves as a mediator between the two. The study addressed the following specific questions and hypotheses: (1) What is the effect of goal setting difficulty on athlete self-regulation in table tennis?; (2) What is the effect of goal setting difficulty on serving success in table tennis?; (3) Does self-regulation mediate the relationship between goal setting difficulty and athletic performance? Given all the research on the fantasy realization theory, we expect that goal setting difficulty will improve athletes’ self-regulation. Given goal setting theory and previous research findings, we expect that the contribution of moderately difficult goals on athletic performance will be higher than that of hard goals and easy goals. Given the self-regulatory model, we expect that the relationship between goal setting difficulty and athletic performance will be mediated either partially or fully by self-regulation.

## Method

### Participants

The sample consisted of 60 female table tennis athletes recruited from a sport school in China. Institutional Review Board approval was not received for this study, as such a process was not in place within China at the time of data collection. However, understanding the importance of this process, the authors ensured that the participants were clearly informed that their participation was voluntary and informed consent was obtained from the school and the parents prior to data collection. The athletes were randomly assigned into an experimental group (n = 30) and a control group (n = 30). The participants in the experimental group had a mean age of 12.9 ± 2.5 years, and average duration of their specialized training in table tennis was 6.8 ± 0.6 years. The mean age of the participants in the control group was 13.6 ± 2.78 years; and average duration of their specialized training in table tennis was 6.5 ± 0.7 years. The decision to recruit juniors rather than elite athletes was based on the following considerations: 1) It is difficult to recruit large numbers of elite athletes, 2) juniors are more accepting of the assigned goals, and were still working on developing the serving skill that was assessed in this study, 3) it is more reasonable to assume that the researchers could modify the self-regulation ability of juniors, and 4) the athletes in this study were all high level table tennis competitors at an elite sports school in China.

#### Task and Measurement

The study used serving success (forehand backspin service, the most basic and critical serving skill in table tennis) within a one minute period as the task; and the “Athlete’s Self-regulation in Motor Learning” (Liu and Zhou, 2007) as the measurement tool, which is a 98-item questionnaire with eight subscales: Planning (e.g., Before practice, I always set specified goals to check the progress and effect), Preparation (e.g., Before practice, I always try to maintain a high level of energy), Consciousness (e.g., During practice, I am aware of my weaknesses), Method (e.g., During practice, I always perform the skill using imagery, imitating, connection, and correcting), Execution (e.g., I will not break my original training plan unless it’s absolutely necessary), Feedback (e.g., After practice, I always verify the degree of my mastery), Remediation (e.g., After practice, I always correct my incorrect movements), and Summarizing (e.g., After practice, I always explore and summarize my technical actions and strategies). The 6-point response scale ranged from 1 (strongly disagree) to 6 (strongly agree). By summing the scores, a global self-regulation score was derived to represent the individual’s overall self-regulation ability. The higher the score, the better the individuals’ self-regulation. Internal coefficient α’s for the subscales ranged from 0.71 to 0.82 (M = 0.76); and stability coefficients α’s for the subscales ranged from 0.75 to 0.93 (M = 0.87) over a 4-week period (Liu and Zhou, 2007).

#### Design

The experiment was designed as a 3 (serving frequency: 20/min - over 75% of participants could achieve, 23/min - over 50% of participants could achieve, and 26/min - less than 10% participants could achieve) × 2 (serving placement: left “small triangle”, and right “small triangle”) model. One definition to point out is that “small triangle” refers to the triangle circled by the 1/4 net line -side line zone, 1/2 side line on half-table - net zone, and the linking line between the two. There are two “small triangles” on each half-table, which are referred to as the left “small triangle” and right “small triangle” (Su, 2003).

#### Procedures

Self-regulation of all participants and serving success (forehand backspin service) within a one minute period were tested at the start of the experiment. Scores from the “Athlete’s Self-regulation in Motor Learning” were used as the foundational value of self-regulation; primary serving success (within the area of “small triangles”) was assessed through three consecutive testings.

The experimental group then practiced in different goal setting conditions, while the control group practiced as normal (without goal setting). Participants in the experimental group were randomly assigned into six different combined goal groups with different servering frequencies and serving placements (5 participants in each group; 20/23/26 serves into left “small triangle”, and 20/23/26 serves into right “small triangle”). During the intervention, they received feedback informing them if they were serving at an appropriate speed to reach the assigned goal or not. The control group was trained by the same coach and completed the same quantity of practice. The intervention period lasted eight weeks (20 minutes, three times per week). Following the end of the intervention period, self-regulation and serving success were retested using the same procedure as the pretest.

#### Data analyses

The T-test and Chi-Square analyses were used to compare the differences of self-regulation and serving success before and after the goal setting intervention. Multivariate analysis of variance and a two-way ANOVA were conducted respectively to test the effect of goal setting difficulty on self-regulation and serving success; and finally, Regression analysis using bootstrapping methods was performed to assess the relationship among goal setting difficulty, self-regulation, and serving success.

## Results

### Effect of Goal Setting Difficulty on Self-Regulation

T-test results showed that there were no significant differences in the eight dimensions or the global self-regulation between the experimental and control groups before the intervention, or between the pre- and post-intervention for the control group. After the intervention (Table 1), all but two dimensions (Consciousness and Execution) for the self-regulation of the experimental group were significantly higher than that of the control group (p < 0.05 for the dimensions of Preparation and Summarizing; p < 0.01 for the dimensions of Planning and Remediation; p < 0.001 for the dimensions of Method, Feedback, and the global self-regulation). For the experimental group (Table 2), all but two dimensions (Consciousness and Remediation) of self-regulation before the experiment were significantly lower than after the experiment (p < 0.05 for the dimensions of Preparation, Method, and Execution; p < 0.01 for the dimension of Feedback; p < 0.001 for the dimensions of Planning, Summarizing, and the global self-regulation).

In addition, a multivariate analysis of variance (Table 3) showed that there was no interaction between serving frequency and serving placement on athletes’ self-regulation (by Hotelling’s criterion: F (5, 24) = 3.398, p > 0.05); serving placement had no main effect on athletes’ self-regulation (by Hotelling’s criterion: F (5, 24) = 2.784, p > 0.05); and serving frequency had a main effect on the dimensions of Planning (F (5, 24) = 4.327, p < 0.05), Method (F (5, 24) = 9.708, p < 0.01), Feedback (F (5, 24) = 8.105, p < 0.01), Remediation (F (5, 24) = 10.217, p < 0.01), Summarizing (F (5, 24) = 10.172, p < 0.01), and the global self-regulation (F (5, 24) = 12.398, p < 0.01). The follow-up univariate tests on serving frequency indicated that the effect of the moderately difficult goal (23/min) on self-regulation was significantly better than that of the easy goal (20/min: F (2, 27) = 5.361, p < 0.05 for Planning; F (2, 27) = 5.420, p < 0.05 for Method; F (2, 27) = 11.384, p < 0.01 for Remediation; F (2, 27) = 16.537, p < 0.001 for Summarizing; and F (2, 27) = 10.479, p < 0.01 for the global self-regulation) and the hard goal (26/min: F (2, 27) = 14.123, p < 0.01 for Method; F (2, 27) = 12.995, p < 0.01 for Feedback; F (2, 27) = 12.004, p < 0.01 for Remediation; F (2, 27) = 13.502, p < 0.01 for Summarizing; and F (2, 27) = 16.504, p < 0.001 for the global self-regulation). No significant differences existed between the easy goal and the hard goal.

### Effect of Goal Setting Difficulty on Serving Success

**Figure 1:**
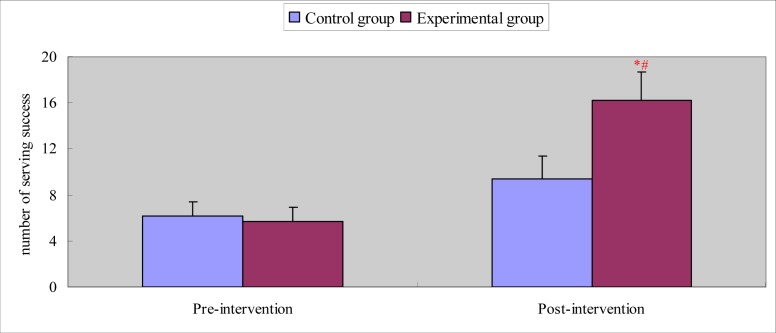
Comparison of serving success after intervention for the experimental and control group * p<0.05, versus Control group; # p<0.05, versus pre-intervention

Chi-Square analyses showed that there was no significant difference in athletes’ serving success between the experimental and control groups before the intervention, or between the pre- and post-intervention for the control group. As shown in Figure 1, after the intervention, athletes’ serving success in the experimental group was significantly better than that of the control group (χ2 = 5.741, p < 0.05); and for the experimental group, athletes’ serving success before the experiment was significantly lower than after the experiment (χ2 = 6.340, p < 0.05).

In addition, results from a two-way ANOVA (Table 4) revealed that there was no interaction between serving frequency and serving placement on athletes’ serving success (F (5, 24) = .685, p > 0.05); serving placement had no main effect on athletes’ serving success (F (5, 24) = 3.716, p > 0.05); and serving frequency had a main effect on athletes’ serving success (F (5, 24) = 37.601, p < 0.001). The follow-up univariate tests on serving frequency indicated that the effect of the moderately difficult goal (23/min) on serving success was significantly better than that of the easy goal (20/min: F (2, 27) = 4.892, p < 0.05) and the hard goal (26/min: F (2, 27) = 8.715, p < 0.01). No significant differences between the easy goal and the hard goal were found (F (2, 27) = 3.243, p > 0.05).

### Relationship between Self-Regulation and Serving Success of Table Tennis Athletes

**Figure 2:**
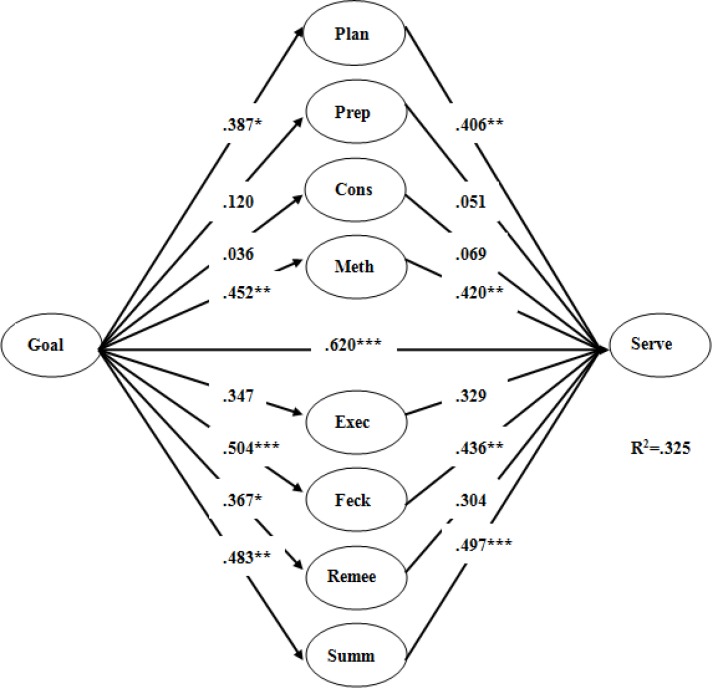
Coefficients representing effects of goal setting difficulty on self-regulation mediators and athletic performance

The results obtained from using Preacher and Hayes’ (2008) bootstrapping procedure showed that the direct effect of goal setting difficulty on serving success was significant (β = 0.620, t = 5.967, SE = 0.07, p < 0.001), and the total effect of goal setting difficulty on serving success was also significant (β =.561, t = 4.396, SE = 0.07, p < 0.001). As Figure 2 shows, goal setting difficulty was positively associated with Planning (β = 0.387, p < 0.05), Method (β =.452, p < 0.01), Feedback (β = 0.504, p < 0.001), Remediation (β = 0.367, p < 0.05), and Summarizing (β = 0.483, p < 0.01). Among the self-regulation variables, Planning (β = 0.406, p < 0.01), Method (β = 0.420, p < 0.01), Feedback (β = 0.436, p < 0.01), and Summarizing (β = 0.497, p < 0.001) had significant direct effects on serving success. The effect of goal setting difficulty on serving success was mediated by Planning (indirect effect = 0.11, p < 0.05), Method (indirect effect = 0.14, p < 0.01), Feedback (indirect effect = 0.21, p < 0.001), and Summarizing (indirect effect = 0.23, p < 0.001). The pair-wise contrasts among these indirect effects were non-significant, indicating that the magnitude of these effects is comparable. Overall, the multiple mediator model was significant (F (9, 20) = 19.317, p < 0.001), accounting for 32.5% of the variance in serving success (Adj. R2 = 0.283).

## Discussion

The purpose of the current study was to verify the effect of goal setting difficulty on serving success in table tennis, and determine if self-regulation is a mediator variable between the two. Results of this study extend our understanding of goal setting theory by demonstrating the facilitation of goal difficulty to the serving success of table tennis athletes. In addition, the psychological mechanism of goal setting on athletic performance was demonstrated through the function of self-regulation as a mediator variable between the two. The findings will be discussed in terms of the following general research questions.

### Effect of Goal Setting Difficulty on Self-Regulation

According to the study by Barry and Stewart (1997), the process of goal setting should involve all dimensions of self-regulation. The present study provided evidence for the effect of goal setting difficulty on self-regulation in table tennis athletes. Goal setting can also be analyzed from a self-regulatory perspective. Successful goal attainment requires the completion of two different tasks. First, athletes must turn their desires into binding goals, and second they have to work to attain those goals. Both of these tasks are believed to benefit from self-regulatory strategies (Oettingen et al., 2001). The manner in which people appraise their goals and the means by which self-regulation guides them toward their goals provides useful context-specific information and also helps improve the motivation that energizes performance (Caprara and Cervone, 2000; Lee et al., 2003; Little, 1983; 1999; McGregor and Little, 1998; Morf, 2002; Pervin, 2003). Karoly and colleague (Karoly, 1991; 1993b; 1999; Karoly and Ruehlman, 1995; 1996) adopted a control systems perspective (e.g., Ford, 1987) which suggests that, if any significant goal is to be reached, the person must be capable of developing and putting in place specific regulatory strategies that include developing an outcome goal, planning, monitoring, taking in feedback, and comparing current activities against other standard(s) of performance.

### Effect of Goal Setting Difficulty on Serving Success

The current results provided evidence for the effect of goal setting difficulty on serving success on table tennis athletes. This result was consistent with goal setting theory (Locke and Latham, 1994), which purports that human activity is purposeful, and guided by conscious goals. Although researchers have employed two ways of explaining how goals influence behavior, research has centered on the direct mechanistic view (as opposed to the indirect thought-process view), which specifies that goals influence performance in one of four direct ways (Locke and Latham, 1985): goals direct attention to important elements of the skill being performed; goals mobilize performer efforts; goals prolong performance persistence; and goals foster the development of new learning strategies.

According to Locke et al. (1989), difficulty is one of the two essential properties of goals (the other is clarity). In the domain of goal setting, the effect of moderately difficult goals and hard goals on athletic performance has had much attention paid to it. Goal setting theory suggests that specific and moderately difficult goals contribute to performance improvement (Locke and Latham, 1990). The current study provided further evidence for the efficacy of moderately difficult goals on athletes’ best performances.

Self-Regulation as a Mediator of Goal Setting Difficulty on Serving Success

To examine the proposed mediational model, Preacher and Hayes’s (2008) bootstrapping procedure was utilized in the present study. Bootstrapping, a non-parametric multiple re-sampling procedure, was used to estimate the size of indirect effects using adjusted percentile (asymmetrical) confidence intervals, and has been shown to function well with simulated data sets (MacKinnon et al., 2004). This procedure is particularly advantageous when applied to the case of multiple mediation, as it helps to determine if an indirect effect exists, and which mediators contribute meaningfully to that effect (Devereux, et al., 2008; Lutz et al., 2008). Bootstrapping can provide an estimate of the individualized indirect effects and contrasts between these indirect effects without problems related to collinearity (Lutz et al., 2008). Preacher and Hayes (2008) created a macro for SPSS that provides an evaluation of the indirect effect of each putative mediated effect and associated bias corrected and accelerated confidence intervals for this effect.

The analysis in the present study was performed using Preacher and Hayes’ SPSS macro (Preacher and Hayes, 2008) and re-sampling 1000 times for the bootstrapping estimates. Ninety-five percent confidence intervals were adjusted for bias, and contrasts between all significant indirect effects were tested. Given the results of the experiment, serving frequency was the only variable applied in the analysis. The findings of the present study indicated that self-regulation may act as a partial mediator of goal setting difficulty on serving success in table tennis, which is clearly congruent with the social cognitive theory (Bandura, 1999) and self-regulatory model (Bandura and Wood, 1989), both of which propose that goal setting is a necessary component of a complex mechanism of self-regulation that helps to increase performance. Within self-regulation, four dimensions contributed to the prediction of serving success-Planning, Method, Feedback, and Summarizing. While the results of this study indicated that self-regulation partially mediated the effect of goal setting difficulty on serving success, self-regulation only accounted for 32.5% of the variance in serving success. Therefore, these self-regulatory skills deserve greater attention in future research in the sport domain.

As a strategy for self-regulation, self-monitoring is believed to impact action in at least two ways (Karoly, 2005). As reported by Lutz et al. (2008), these impacts are “First, self-monitoring serves an informational or record-keeping function, providing persons pursuing a change goal with the opportunity to systematically track their to-be-altered performance. Second, self-monitoring can be therapeutic or change inducing when individuals engaged in self-recording become more mindful of the undesirable or desirable aspects of the monitored actions, and subsequently undertake the initial steps toward habit change.” In fact, the role of consistent self-monitoring has been confirmed in research examining other difficult self-regulatory challenges (e.g., weight loss through dietary restraint, Boutelle and Kirschenbaum, 1998). Taking into account the social cognitive theory, the self-regulatory model, and the current results, self-regulation can be considered as the partial mediator between goal setting and athletic performance. Therefore, beyond the use of goal setting, it would be wise to also help athletes develop their self-regulatory abilities.

### Limitations & Future Directions

Although the present study provides a snapshot of the mediating mechanism between goal setting and athletic performances, it should be acknowledged that several salient limitations apply. First, the goals in the current study were set by the researcher (i.e., assigned goals, based upon group performance levels). According to Button et al. (1996), assigned goals influence personal goals through goal acceptance and commitment. They argue that individuals adopt the externally set goals as their own and become committed to attaining them. However, much of the literature in this area indicates that the effect of self-set goals (set by the participants) on athletic performances was better than that of assigned goals (Li et al., 1996). Second, all the participants in the current study were female athletes. Research has shown that girls have a higher degree of self-regulation (Song and Qi, 2003). Third, the sample size in the present study was relatively small. Kyllo and Landers (1995) and Burton and Naylor (2002) suggested that research designs utilizing relatively small sample sizes could be partially responsible for the inconsistent results in goal setting literature within sport/exercise settings.

Despite these limitations, the current study aptly demonstrated the effect of goal setting on athletic performance in junior athletes and the potential mediating mechanism between the two. Future research should attempt to establish a model to clarify the potential mediating variables between goal setting and athletic performance, which should be applied into practical settings for performance enhancement. Another point of interest in the future is to confirm and clarify the perceived motivational climate and goal orientations of the athletes, both of which are important factors influencing performance (Bueno et al., 2008). Moreover, both the negative states (Conroy et al., 2002; Lazarus, 1999; Lewthwaite, 1990) and positive states (Jackson and Roberts, 1992; Pates et al., 2001) of athletes affect their performance. In fact, our previous research has indicated that both trait flow and state flow can be considered as intermediate variables between goal setting and performance. Therefore, it would be interesting to study the relationships between motivational climate, goal orientations, emotional states, and performance in future research. Future researchers may also consider using movement analysis systems to analyze the serving movements of the athletes. Finally, it is important that future researchers study the self-regulation model with regard to helping athletes achieve their goals, and to improve their athletic performance.

## Figures and Tables

**Table 1:** Comparison of self-regulation after the intervention

Dimensions	Experimental group		Control Group		t	p

	M	SD	M	SD		
Planning	42.50	4.42	36.77	4.90	3.058	0.004^**^
Preparation	32.67	1.73	29.83	3.49	2.217	0.047^*^
Consciousness	37.67	5.71	35.53	4.58	1.595	0.116
Method	222.33	4.69	176.77	17.31	10.621	0.000^***^
Execution	38.83	3.59	36.83	3.64	0.678	0.500
Feedback	53.67	2.66	46.57	3.22	6.127	0.000^***^
Remediation	53.77	3.41	49.20	3.62	3.274	0.003^**^
Summarizing	39.33	1.83	35.83	2.61	2.894	0.023^*^
Global	520.77	26.33	466.73	31.79	10.222	0.000^***^

p< 0.05.

** p < 0.01.

*** p < 0.001.

**Table 2:** Comparison of self-regulation for the experimental group

Dimensions	Pre-intervention		Post-intervention		t	p

	M	SD	M	SD		
Planning	36.77	5.53	42.50	4.42	−3.851	0.001^**^
Preparation	30.03	2.34	32.67	1.73	−2.967	0.017^*^
Consciousness	35.00	6.95	37.67	5.71	−1.652	0.109
Method	203.60	8.32	222.33	4.69	−2.436	0.021^*^
Execution	36.73	4.21	38.83	3.59	−2.099	0.045^*^
Feedback	44.83	2.89	53.67	2.66	−3.473	0.009^**^
Remediation	47.63	3.42	53.77	3.41	−1.407	0.247
Summarizing	33.20	2.06	39.33	1.83	−4.214	0.001^***^
Global	467.79	22.26	520.77	26.33	−6.617	0.001^***^

**Table 3:** Effect of different goal setting difficulties on self-regulation (F value)

DV	Plan	Prep	Cons	Meth	Exec	Feck	Reme	Summ	Global
IV									
Frequency	4.327^*^	1.137	0.135	9.708^**^	0.356	8.105^**^	10.217^**^	10.172^**^	12.398^**^
Placement	4.206	1.218	0.145	1.802	0.272	4.103	3.791	3.381	3.158
Frequency × Placement	0.866	0.639	0.086	0.391	0.004	1.666	0.970	2.697	0.786

**Table 4:** Effect of different goal setting difficulties on serving success

Source	SS	DF	MS	F	P
Frequency	0.056	2	0.028	37.601	0.001^***^
Placement	0.005	1	0.005	3.716	0.057
Frequency × Placement	0.001	2	0.001	0.685	0.514
